# Cisplatin plus paclitaxel chemotherapy with or without bevacizumab in postmenopausal women with previously untreated advanced cervical cancer: a retrospective study

**DOI:** 10.1186/s12885-021-07869-7

**Published:** 2021-02-06

**Authors:** Guanghua Chu, Xiangzhen Liu, Weiguang Yu, Meiji Chen, Lingyun Dong

**Affiliations:** 1 Department of Gynecology, Northwest Women’s and Children’s Hospital, No. 1616, Yanxiang Road, Qujiang New District, Xi’an, 710061 Shaanxi China; 2 Department of Oral and Maxillofacial Surgery, The First Affiliated Hospital, Sun Yat-sen University, No. 58, Zhongshan 2nd Road, Yuexiu District, Guangzhou, 510080 China; 3 Department of Orthopedics, The First Affiliated Hospital, Sun Yat-sen University, No. 58, Zhongshan 2nd Road, Yuexiu District, Guangzhou, 510080 China; 4 Department of Pediatrics, The First Affiliated Hospital, Sun Yat-sen University, No. 58, Zhongshan 2nd Road, Yuexiu District, Guangzhou, 510080 China; 5 Department of Gynecology and obstetrics, Shanghai Public Health Clinical Center, No. 2901 Caolang Road, Jinshan District, Shanghai, 201508 China

**Keywords:** Bevacizumab, Advanced, Cervical cancer, Chemotherapy, Survival

## Abstract

**Background:**

The aim of this study was to assess the survival outcomes of cisplatin-paclitaxel chemotherapy plus bevacizumab (CPB) versus cisplatin-paclitaxel chemotherapy alone (CPA) in postmenopausal women with previously untreated advanced cervical cancer (CC).

**Methods:**

Consecutive postmenopausal women who experienced CPB or CPA were identified retrospectively from our medical centre during 2015–2019. Follow-up visits occurred 1 and 3 months after starting CPB or CPA. Afterwards, this assessment was conducted every 3 months for 1 year and then yearly thereafter. The primary endpoints were overall survival (OS) and progression-free survival (PFS); secondary endpoints were the frequency and severity of adverse events (AEs).

**Results:**

Two hundred forty-six postmenopausal women were included (CPB, *n* = 124; CPA, *n* = 122). The median follow-up for the entire cohort was 24 months (range, 2–32). At the final follow-up, a significant difference was detected in terms of median OS (16.4 months [95% CI, 15.3–17.1] for CPB vs. 12.3 months [95% CI, 10.2–13.5] for CPA; hazard ratio (HR) 0.69, 95% CI, 0.49–0.99; *p* = 0.001), and the median PFS was longer in the CPB group than in the CPA group (9.2 months [95% CI, 8.3–10.7] vs. 7.9 months (95% CI, 6.1–8.6) (HR 0.62, 95% CI, 0.47–0.82; *p* < 0.001). There were significant differences in the number of AEs between the groups (hypertension grade ≥ 2 [*p* < 0.001], neutropenia grade ≥ 4 [*p* < 0.001], and thrombosis/embolism grade ≥ 3 [*p* = 0.030]).

**Conclusions:**

Among postmenopausal women with previously untreated advanced CC, those who received CPB experienced superior survival benefits compared to those who received CPA. The safety profile for CPB was controllable despite the long duration of CPB use.

## Background

Advanced cervical cancer (CC) continues to be an important cause of mortality among women [1–3]. The management of recurrent, persistent, or metastatic CC remains challenging, as evidenced by previous trials [1, 4, 5]. The majority of these patients receive concurrent cisplatin-based chemotherapy as the primary treatment option [6]. A newly approved regimen, the addition of bevacizumab (BEV) to combination chemotherapy, has been shown to improve survival in patients with advanced CC [1, 5]. Findings from a randomised, controlled, open-label, phase 3 trial (Gynecologic Oncology Group 240) [1] indicate that cisplatin-paclitaxel plus BEV markedly improves median overall survival (OS) compared to cisplatin-paclitaxel chemotherapy alone in advanced CC (16.8 months vs. 13.3 months, respectively; hazard ratio [HR] 0.77, 95% CI, 0.62–0.95; *p* = 0.007). Furthermore, the survival benefit conferred by the incorporation of BEV into cisplatin-paclitaxel chemotherapy tends to be sustained with extended follow-up as evidenced by the OS curves remaining separated. However, in the phase 3 trial, it was unclear whether the selected cohort of patients had previously received chemotherapy. Moreover, these study participants were not restricted to postmenopausal women.

In 2004, BEV was approved by the United States Food and Drug Administration for specific types of cancer, and became the first antiangiogenic agent to be used in several chemotherapy regimens [1, 7, 8]. BEV is a highly purified recombinant human monoclonal IgG1 antibody, and inhibits angiogenesis by binding and neutralizing circulating vascular endothelial-derived growth factor (VEGF) [1, 7, 9]. It can inhibit tumour growth and prolong the survival of patients with advanced CC [10]. Nevertheless, the number of reports on specific groups of female patients is extremely limited [11]. Whether the previous conclusions also apply to postmenopausal women with previously untreated advanced CC remains unclear.

Herein, we performed a retrospective study to verify whether postmenopausal women with previously untreated advanced CC who received cisplatin-paclitaxel chemotherapy plus BEV (CPB) had a greater survival advantage than those who received cisplatin-paclitaxel chemotherapy alone (CPA).

## Methods

### Data

Consecutive postmenopausal women with previously untreated advanced CC who received CPB or CPA between January 2015 and August 2019 and for whom baseline data were available at the present analysis were identified from the First Affiliated Hospital, Sun Yat-sen University. Inclusion criteria were as follows: Chinese population; postmenopausal women with previously untreated histologically proven CC stage IV/B; advanced CC was confirmed by our institutional pathology laboratory and clinical imaging data according to FIGO 2018 cervical cancer staging criteria [12]; measurable disease; and a GOG performance status score of 0 or 1(0: fully active; 1: physically strenuous activities but ambulatory). Key exclusion criteria were as follows: prior use of targeted drug(s); previous chemotherapy, radiotherapy, chemoradiotherapy, or surgery for advanced CC; CPB or CPA discontinuation, regardless of the drug-induced adverse events (AEs); symptomatic brain metastases; cachexia; severe medical illness (such as, uncontrolled infection or hypertension, hypertension with multiple complications, HIV infection); surgical emergency (such as, intestinal obstruction); key organ function failure (such as, lung, brain, kidney, and/or heart); active bleeding conditions or coagulopathy; arterial or venous thrombosis; dementia or psychiatric disorders; and collagenosis.

### Study design and treatment

A retrospective, single-centre study was conducted in which eligible postmenopausal women had underwent intravenous CPB or CPA regimen [1]. The CPB treatment consisted of cisplatin (50 mg/m2 on day 1) plus paclitaxel (175 mg/m2 on day 1) plus BEV (15 mg/kg on day 1). The CPA treatment consisted of cisplatin (50 mg/m2/day) plus paclitaxel (175 mg/m2/day). The regimens for CPB and CPA were repeated every 21 days until disease progression, death, or intolerable toxic effects. The management of hypertension in patients with advanced CC receiving BEV was consistent with previous recommendations reported by Plummer et al. [13].

### Outcomes and assessments

The primary endpoints were overall survival (OS) and progression-free survival (PFS); secondary endpoints were the frequency and severity of AEs. OS was defined as the date form drug treatment to death from any cause or date last seen. PFS was defined as the date from drug treatment to the date of either disease progression or death from any cause. Postmenopausal women were defined as female patients who had not menstruated for at least a year, excluding menopause due to related diseases (i.e., endometriosis, endocrine disorders). Disease progression was defined according to the Response Evaluation Criteria in Solid Tumours (RECIST, version 1.1) [14]. Computed tomography (CT) was carried out at each follow-up. Safety was assessed using the National Cancer Institute Common Terminology Criteria for Adverse Events, version 4.0 [15]. Persistent disease was defined as not achieving a complete clinical response [8]. Pain was assessed using the Brief Pain Inventory [16]. Details related to pain and hypertension (occurrence, duration, and severity) were registered. Follow-up time was calculated from the date of clinical staging to the date last seen. Follow-up visits occurred 1 and 3 months after starting CPB or CPA. Afterwards, this assessment was conducted every 3 months for 1 year and then yearly thereafter.

### Statistical analysis

Categorical variables were compared using Chi-square tests; continuous variables were compared using Student’s t-test for normally distributed variables and Mann-Whitney U test for non-normally distributed variables. Survivorship curves were completed by means of the Kaplan-Meier methods with a log-rank test. The acquisition of the hazard ratio (HR) and confidence interval (CI) were achieved through a stratified log-rank test. All statistical tests used a 2-tailed of 0.05. The survival curves were made using GraphPad Prism 8.0. To perform other statistical analysis, SPSS 26.0 (IBM, Inc., NY, America) was used.

## Results

### Demographic characteristics

**Fig. 1:**
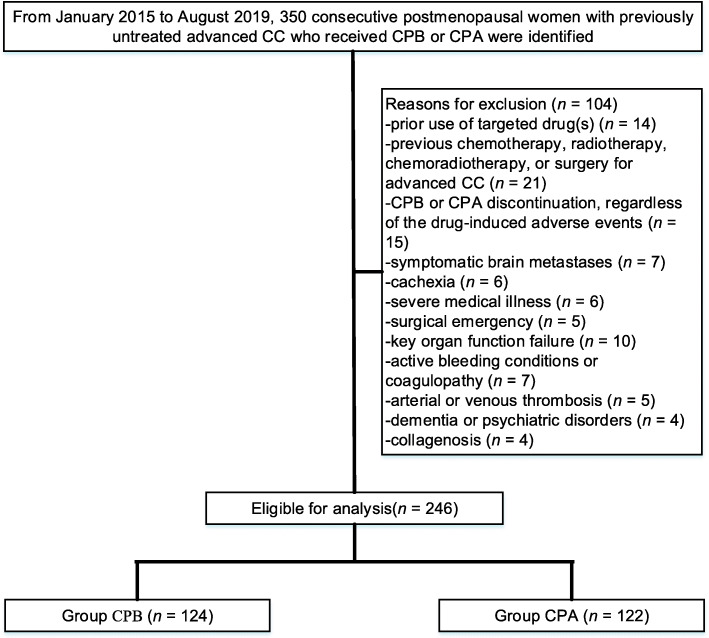
Flow diagram exhibiting the methods applied to identify objects to evaluate survival of cisplatin-paclitaxel chemotherapy plus BEV (CPB) versus cisplatin-paclitaxel chemotherapy alone (CPA) in postmenopausal women with previously untreated advanced cervical cancer (CC)

We identified 350 postmenopausal women with previously untreated advanced CC, of whom 246 postmenopausal women were included. Of these 246 women, 124 received CPB, and 122 received CPA (Fig. 1). Table 1 showed the characteristics of postmenopausal women who underwent CPB versus CPA. The mean age was 62.1[±7.59] years for CPB and 62.2[±6.84] years for CPA. ECOG performance status was 0 in 37.1% of patients and 1 in 62.9% of patients receiving CPB versus 0 in 34.4% of patients and 1 in 65.6% of patients receiving CPA (*p* = 0.663). Statistically significant differences were not detected with respect to age, body mass index (BMI), haemoglobin < 11 g/dL, histology, IV/B CC, duration of drug, GOG performance status, and number of metastatic sites. The median follow-up for the entire cohort was 24 months (range, 2–32). The median number of drug cycles for individuals undergoing CPB was 8 (range, 1–26), and for those who underwent CPA, the median was 7 (range, 1–28). Of 246, 197 (80.1%) individuals discontinued CPB or CPA, primarily attributed to disease progression (55.8% for CPB vs. 44.2% for CPA, *p* = 0.001). Even though more patients developed disease progression in the CPB group but there was a significant delay in the time taken for disease to progress in this group which contributed to significantly longer PFS.

**Table 1:** Patient demographics between groups

Variable	CPB (n = 124)	CPA (n = 122)	*p*-value
Age (years)	62.1 ± 7.59	62.2 ± 6.84	0.183^*a*^
BMI	24.5 ± 5.31	25.6 ± 6.19	0.102^*a*^
Haemoglobin < 11 g/dL^c^, n (%)	36 (29.0)	32 (26.2)	0.624^*b*^
Histology, n (%)			0.439^*b*^
Squamous	79 (63.7)	72 (59.0)	
Adenocarcinoma	34 (27.4)	37 (30.3)	
Adenosquamous	11 (8.9)	13 (10.7)	
IVB cervical cancer, n (%)			0.533^*b*^
Asymptomatic CNS metastasis	45 (36.3)	49 (40.2)	
Without CNS metastasis	79 (63.7)	73 (59.8)	
Duration of drug (months)	17.8 ± 7.46	17.4 ± 7.63	0.219^*a*^
ECOG status, n (%)			0.663^*b*^
0^d^	46 (37.1)	42 (34.4)	
1^e^	78 (62.9)	80 (65.6)	
GOG status^f^, n (%)			0.672^*b*^
0^f^	49 (39.5)	45 (36.9)	
1^g^	75 (60.5)	77 (63.1)	
No. of metastatic sites, n (%)			0.445^*b*^
3	38 (30.6)	30 (27.8)	
> 3	71 (57.3)	78 (62.0)	
Unknown	15 (12.1)	14 (10.2)	

^a^Analysed using an independent samples t-test; ^b^Analysed using the Mann-Whitney U test;

^c^Haemoglobin level was calculated one week before the start of CPB or CPA; 0^d^, Fully active, able to carry on all pre-disease performance without restriction; 1^e^, Restricted in physically strenuous activity but ambulatory and able to carry out work of a light or sedentary nature, e.g., light housework, office work; 0^f^: fully active; 1^g^: restricted in physically strenuous activities, but ambulatory. *CPB* cisplatin-paclitaxel chemotherapy plus bevacizumab, *CPA* cisplatin-paclitaxel chemotherapy alone, *BMI* body mass index, *CNS* central nervous system, *ECOG* Eastern Collaborative Oncology Group, *GOG* Gynecologic Oncology Group

### Survival analysis

**Fig. 2:**
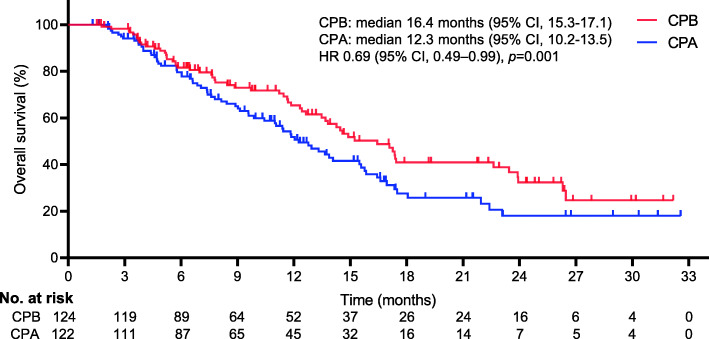
Kaplan-Meier curves for overall survival. The median overall survival was 16.4 months (95% confidence interval [CI], 15.3–17.1) for CPB and 12.3 months (95% CI, 10.2–13.5) for CPA (HR 0.69, 95%CI, 0.49–0.99; *p* = 0.001). *The hazard ratio was calculated using a Cox proportional hazards model, with the age, BMI, haemoglobin < 11 g/dL, histology, IVB CC, duration of drug, performance scores, and number of metastatic sites used as covariates and therapy as the time-dependent factor

**Fig. 3:**
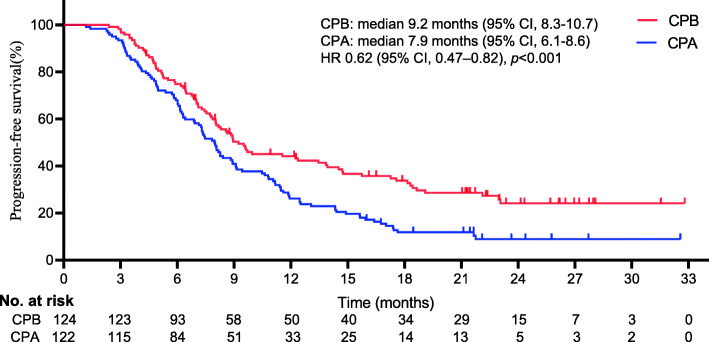
Kaplan-Meier curves for progression-free survival. The median progression-free survival was 9.2 months (95% confidence interval [CI], 8.3–10.7) for CPB and 7.9 months (95% CI 6.1–8.6) for CPA (HR 0.62, 95%CI, 0.47–0.82; *p* < 0.001). *The hazard ratio was calculated using a Cox proportional hazards model, with the age, BMI, haemoglobin < 11 g/dL, histology, IVB CC, duration of drug, performance scores, and number of metastatic sites used as covariates and therapy as the time-dependent factor

Figures 2 and 3 show the KM survival plot for the differences in OS and PFS between the 2 different regimens at the final follow-up, respectively. Throughout the follow-up period, 129 deaths were reported (52% [129/246]; 58 patients for CPB vs. 71 patients for CPA). A borderline noteworthy distinction was detected in the median OS between the two regimens (16.4 months [95% CI, 15.3–17.1] for CPB vs. 12.3 months [95% CI, 10.2–13.5] for CPA). The incorporation of BEV significantly improved the median OS compared with chemotherapy alone (HR 0.69, 95% CI, 0.49–0.99; *p* = 0.001). There was also a distinct difference in the median PFS between the two regimens (9.2 months [95% CI, 8.3–10.7] for CPB vs. 7.9 months for CPA (95% CI, 6.1–8.6) (HR 0.62, 95% CI, 0.47–0.82; *p* < 0.001).

### Adverse events

Of the 246 patients, 43 (25 [20.2%] receiving CPB vs. 18 [14.8%] receiving CPA; *p* = 0.265) had not documented persistent disease at the final follow-up. Table 2 demonstrates the frequency of AEs probably associated with CPB or CPA. Compared with CPB, CPA was associated with fewer AEs (9.9% vs. 12.0%, *p* < 0.001). There was a significant difference between the groups for hypertension grade ≥ 2 (25.0% receiving CPB vs. 4.1% receiving CPA, *p* < 0.001), but it was generally controllable. Additionally, significant differences were noted between the groups for neutropenia grade ≥ 4 (33.9% receiving CPB vs. 14.8% receiving CPA, *p* < 0.001) and thrombosis/embolism grade ≥ 3 (8.9% receiving CB vs. 2.5% receiving CA, *p* = 0.030). There were no significant differences in terms of genitourinary fistula grade ≥ 2, gastrointestinal fistula grade ≥ 2, gastrointestinal bleeding grade ≥ 3, or pain grade ≥ 2.

**Table 2:** Adverse events

Grade, n%	CPB (*n* = 124)	CPA (*n* = 122)	HR (95%)	*p-*value
≥ 2 genitourinary fistula	0 (0)	0 (0)	NA	1.000
≥ 2 gastrointestinal fistula	0 (0)	0 (0)	NA	1.000
≥ 3 gastrointestinal bleeding	0 (0)	0 (0)	NA	1.000
≥ 2 hypertension	31 (25.0)	5 (4.1)	7.00 (4.52–6.02)	< 0.001*
≥ 4 neutropenia	42 (33.9)	18 (14.8)	4.00 (3.83–5.36)	< 0.001*
≥ 3 thrombosis/embolism	11 (8.9)	3 (2.5)	2.00 (1.48–3.71)	0.030*
≥ 2 pain	38 (30.6)	34 (27.9)	3.00 (0.14–4.23)	0.633

*Statistically significant. *CPB* cisplatin-paclitaxel chemotherapy plus bevacizumab, *CPA* cisplatin-paclitaxel chemotherapy alone, *HR* Hazard ratio, *NA* not applicable

## Discussion

Findings from this retrospective study showed that the incorporation of BEV into cisplatin-paclitaxel chemotherapy led to significantly longer PFS times for postmenopausal women with previously untreated advanced CC, leading to significantly longer OS than those who received cisplatin-paclitaxel chemotherapy alone, with a controllable safety profile. The Kaplan–Meier curve for survival among these cases indicated an early advantage for patients receiving CPB that remained until final follow-up, with a difference in median OS of 4.1 months, which reached statistical significance.

The conclusion of the present study is consistent with the findings from a previous phase III trial [8], which assessed the effectiveness of BEV and nonplatinum combination chemotherapy in patients with recurrent, persistent, or metastatic CC. In this trial, the bevacizumab-containing regimen markedly improved the median OS compared to chemotherapy alone (17.0 months vs. 13.3 months; HR for death, 0.71; 98% CI, 0.54–0.95). Other clinical trials [5, 17] investigating the combined use of cisplatin-paclitaxel chemotherapy and the antiangiogenic agent BEV have shown a reduced hazard of disease progression, with median OS times ranging from approximately 16 to 18 months. The combination was effective and well tolerated in patients with advanced CC, which is currently recommended by the National Comprehensive Cancer Network as the standard of care for such patients [10]. The lack of effective therapies in advanced CC following the development of acquired resistance to conventional chemotherapy is a major clinical problem [5, 8]. The prognosis of these patients is still not favourable. Previous chemotherapy regimens have demonstrated a positive effect on metastatic CC; nevertheless, cisplatin-paclitaxel chemotherapy is still regarded as the preferred treatment option, which was established by the GOG 204 protocol [18].

VEGF contributes to the development of new tumour vasculature and is important for the survival and proliferation of cancer cells [10]. The expression of VEGF tends to be correlated with the biological aggressiveness of CC and is associated with poor survival [17, 19, 20]. Sequestration of VEGF using BEV when combined with chemotherapy was associated with improved survival among women with advanced CC [5, 20]. A phase II study (GOG-227C) [21] showed that BEV prevents tumour angiogenesis by blocking VEGF and was demonstrated to be active in recurrent CC. ESMO Clinical Practice Guidelines [22] indicated that the incorporation of BEV into cisplatin-paclitaxel chemotherapy is the preferred first-line regimen in advanced CC based on the balance between efficacy and toxicity profile.

Consistent with the GOG 240 [5], the current study showed that marked separations in median OS were observed (16.4 months [95% CI, 15.3–17.1] for CPB vs. 12.3 months [95% CI, 10.2–13.5] for CPA; HR 0.69, 95% CI, 0.49–0.99; *p* = 0.001). A retrospective study [23] of 52 patients with advanced CC who received the cisplatin-paclitaxel-BEV triplet reported a median OS of 15.3 months and a median PFS of 9.8 months. Recently, a retrospective observational study [24] involving 264 Chinese women with advanced CC who underwent cisplatin-paclitaxel-BEV triplet or cisplatin-paclitaxel chemotherapy alone showed that the cisplatin-paclitaxel-BEV triplet is associated with improved survival compared to cisplatin-paclitaxel chemotherapy alone (median OS: 540 days [95% CI, 483–597] for cisplatin-paclitaxel-BEV triplet vs. 357 days [95% CI, 264–450)] for cisplatin-paclitaxel chemotherapy alone; HR 1.21, 95% CI, 1.14–1.73; *p* = 0.002). A phase 3 trial (GOG 240) [1] showed that the cisplatin-paclitaxel-BEV triplet yields more significant improvement in survival than cisplatin-paclitaxel chemotherapy alone. An NRG Oncology/GOG study [5] of 390 female patients with advanced CC showed that improvements in survival were associated with BEV.

The efficacy of BEV has been revealed to be involved in the reconstruction of normal vasculature at the tumour site, initiating enhanced nutrient and oxygen supply while also escalating the delivery of cisplatin-paclitaxel drugs to the area occupied by the tumour [3, 25, 26]. VEGF signalling participates in several important physiologic processes (i.e., angiogenesis and blood pressure regulation) [3]. While blocking VEGF signalling may inhibit the progression of advanced CC, it can also initiate unintended effects because several other functional adjustments are achieved through VEGF signalling [27, 28]. BEV is directly related to the development of hypertension, which is a recognized effect of anti-VEGF therapy [3]. Since VEGF is indispensable and used to sustain vascular homeostasis, blocking the VEGF pathway can initiate endothelial dysfunction and hypertension [28]. The pathogenesis of this type of BEV-induced hypertension is not fully understood. One possible explanation [13, 29, 30] is that blocking VEGF results in a decrease in nitric oxide (NO) that dilates blood vessels.

Several important limitations should be recognized. First, this retrospective study had inherent shortcomings. Although this study included only postmenopausal women with previously untreated advanced CC, the relevant regression analysis can only control for measurable confounding factors. Residual confusion remains an important issue and may lead to underestimation of the harm associated with drug intervention. Second, when paclitaxel was administered at the maximum dose of 175 mg/m^2^/day, toxicity was escalated to a degree, which may result in a shorter medication cycle. Nonetheless, since the specifications of the drugs used by patients are uniform, this can avoid drug differences in the baseline data. Third, the current subjects were limited to postmenopausal women with previously untreated advanced CC, and thus, the results may not be generalizable to patients receiving routine treatment.

## Conclusion

The results reported here may support the growing body of evidence demonstrating that the cisplatin-paclitaxel-BEV triplet combination is associated with increased survival benefit in postmenopausal women with previously untreated advanced CC. Furthermore, the safety and efficacy of long-term therapy with cisplatin-paclitaxel with and without BEV maintenance needs to be explored in postmenopausal women with previously untreated advanced CC.

## Data Availability

The datasets used and/or analysed during the current study are available from the corresponding author upon reasonable request.

## Abbreviations

CC: Cervical cancer
BEV: Bevacizumab
VEGF: Vascular endothelial growth factor
OS: Overall survival
HR: Hazard ratio
CPB: Cisplatin-paclitaxel chemotherapy plus BEV
CPA: Cisplatin-paclitaxel chemotherapy alone
ECOG: Eastern Collaborative Oncology Group
GOG: Gynecologic Oncology Group
CNS: Central nervous system
PFS: Progression-free survival
BMI: Body mass index
CT: Computed tomography
RECIST: Response Evaluation Criteria in Solid Tumours
CI: Confidence interval
